# Dataset of pharmaceuticals in the Danube and related drinking water wells in the Budapest region

**DOI:** 10.1016/j.dib.2020.106062

**Published:** 2020-07-23

**Authors:** Attila Csaba Kondor, Gergely Jakab, Anna Vancsik, Tibor Filep, József Szeberényi, Lili Szabó, Gábor Maász, Árpád Ferincz, Péter Dobosy, Zoltán Szalai

**Affiliations:** a Geographical Institute, Research Centre for Astronomy and Earth Sciences, MTA Centre for Excellence, Budaörsi út 45., Budapest H-1112, Hungary; b Department of Environmental and Landscape Geography, Eötvös Loránd University, Pázmány Péter sétány 1/C., Budapest H-1117, Hungary; c Institute of Geography and Geoinformatics, University of Miskolc, Egyetemváros, Miskolc H-3515, Hungary; d MTA-Centre for Ecological Research, Balaton Limnological Institute, Klebelsberg Kuno u. 3., Tihany, H-8237, Hungary; e Department of Aquaculture, Szent István University, Páter K. u. 1., Gödöllő, H-2100, Hungary; f MTA-Centre for Ecological Research, Danube Research Institute, Karolina út 29., Budapest, H-1113, Hungary

**Keywords:** Drinking water, Danube, Organic micropollutants, Persistency

## Abstract

The present dataset provides data on the pharmaceutically active compounds (PhACs) concentrations measured in the Danube and the drinking water abstraction wells (DWAW) in the Budapest region. Grab samples were collected during five periods. One hundred and seven water samples from the Danube and ninety water samples from the relevant DWAWs were analyzed to quantify physical-chemical parameters, trace element concentrations, and one hundred and eleven PhACs, including pharmaceutical derivatives, illicit drugs, and alkaloids. The ion concentrations were measured using dual channel ion chromatography, spectrophotometric and titrimetric methods, and inductively coupled plasma mass spectrometry. PhACs concentrations were measured after solid-phase extraction applying supercritical fluid chromatography coupled with tandem mass spectrometry. Fifty-two PhACs were quantified in the Danube, and ten PhACs were present in >80% of the samples. Whereas thirty-two PhACs were quantified in the DWAWs. The present dataset is useful for further comparisons and meta-analyses.

Specifications Table

**Table:** 

**Subject**	Pollution
**Specific subject area**	Environmental pollution in surface waters
**Type of data**	Table
**How data were acquired**	Using dual channel ion chromatography (Dionex ICS 5000+, Thermo Fischer Scientific, USA); spectrophotometric and titrimetric methods; inductively coupled plasma mass-spectrometry (Plasma Quant Elite, Analytik Jena, Jena, Germany); Solid-phase extraction system(AutoTrace 280 SPE instrument; Thermo Scientific, USA); supercritical fluid chromatography coupled with tandem mass spectrometry (ACQUITY UPC2 system, Waters and Xevo TQ-S Triple Quadrupole, Waters Corporation, UK)
**Data format**	Raw Analyzed
**Parameters for data collection**	One hundred and seven water samples from the Danube and ninety water samples from the relevant drinking water abstraction wells were collected as grab samples in five periods (07.2017–11.2018) at the Budapest region.
**Description of data collection**	Altogether one hundred and ninety-seven water samples were analyzed to quantify general water chemistry, trace element concentrations, and one hundred and eleven pharmaceutically active compounds including pharmaceutical derivatives, hormones, illicit drugs, and alkaloids.
**Data source location**	Institution: Geographical Institute, Research Centre for Astronomy and Earth Sciences, MTA Centre for Excellence City: Budapest Country: Hungary Latitude and longitude: N 47.2444–47.8193° E 18.9160–19.1358°
**Data accessibility**	With the article
**Related research article**	A.Cs. Kondor, G. Jakab, A. Vancsik, T. Filep, J. Szeberényi, L. Szabó, G. Maász, Á. Ferincz, P. Dobosy, Z. Szalai, Occurrence of pharmaceuticals in the Danube and drinking water wells: Efficiency of riverbank filtration, Env. Poll. 265, 114,893, https://doi.org/10.1016/j.envpol.2020.114893

## Value of the Data

• Scientists studying water chemistry and pollution degree of the river Danube can benefit the data. Engineers of water supply facilities can also use the data.
• The database provides a stable base for the modeling of bank filtration or the calibration/validation for existing models.
• The database can contribute to a safer drinking water supply for the Budapest Metropolitan Region.

## 1 Data description

The measured general physical-chemical parameters and PhACs concentrations of the Danube samples are given in Table S1. The same properties for the drinking water abstraction well (DWAW) samples are presented in Table S2. From the investigated 111 PhACs, 52 were found in the Danube, and 32 in the DWAWs. In the Danube, 10 PhACs were present in >80% of the samples, whereas carbamazepine was found in 106 Danube samples. In contrast, 10% of the samples were free from carbamazepine in the DWAWs [1].

## 2 Experimental design, materials and methods

### 2.1 Sampling

During the five sampling periods (29.06.2017 – 19.11.2018), 107 water samples were collected from the Danube between 1600 and 1700 river kilometers using a boat. Each sampling campaign was carried out on low water discharge: three campaigns in the summer and two campaigns in winter. The DWAWs were also sampled during the same five periods with a ten days delay. Altogether, 90 samples were collected from the wells through sampling taps. Table S1 and Table S2 provide the locations of the sampling points at the Danube River and the DWAWs respectively.

### 2.2 Measurement of general water chemistry

Turbidity, pH, dissolved oxygen concentration, electric conductivity as well as temperature were recorded directly from the water with a turbidimeter (VWR International, USA) and a Hanna Multi Meter (Hanna Instrument, USA). A Dionex ICS 5000+ dual channel ion chromatography system (Thermo Fischer Scientific, USA) was applied to determine the calcium, sodium, ammonium, potassium, and magnesium, as well as nitrate, chloride, fluoride, bromide and sulfate concentrations, whereas a Multi N/C 3100 TC–TN analyzer (Analytik Jena, Germany) was used to quantify total organic carbon and total nitrogen concentrations. The trace element concentrations were determined using a PlasmaQuant MS Elite inductively coupled plasma mass-spectrometer (Analytik Jena, Germany). Titrimetry and spectrophotometry were applied to measure total hardness, alkalinity, phosphate, and nitrite concentrations. Additional properties are presented by Jakab et al. [2].

### 2.3 PhACs quantification

Water samples were acidified (formic acid) and spiked with the corresponding mass-labeled internal standard (IS) for quantification. Analytes in the filtered samples were isolated by an AutoTrace 280 automata solid-phase extraction system (Thermo Scientific) using Strata X-CW cartridges (#8B-S035-FCH, Phenomenex) because of the low concentration. To achieve the required sensitivity, steroid agents were derivatized with dansyl-chloride. Supercritical fluid chromatography (ACQUITY UPC2 system, Waters) coupled with tandem mass spectrometry (MS/MS) (Xevo TQ-S Triple Quadrupole, Waters) was applied to analyze and quantify the drug residuals. MassLynx software (V4.1 SCN950) was used for recording data in triplicate, whereas the evaluation was carried out with TargetLynx XS software. The separation of target molecules was performed on an ACQUITY UPC2 BEH analytical column (#186,007,607, Waters). The electrospray ionization was provided by a spray voltage of 3 kV in both ion modes. All MS/MS measurements were completed in multiple-reaction-monitoring mode, more details are presented by Jakab et al. [2] and Maasz et al. [3]. The observed ions (mass in *m/z*) were accepted and quantified if they met the following criteria: retention time, proper MS1 mass, MS2 masses, IS correction, and fragmentation pattern. The method characteristics, limits of detection, and quantification values are given by Kondor et al. [1].

## Declaration of Competing Interest

The authors declare that they have no known competing financial interests or personal relationships which have, or could be perceived to have, influenced the work reported in this article.

## Appendix Supplementary materials

Image, application 1
